# Peoria District Medical Society, Illinois

**Published:** 1848-10

**Authors:** 


*Peoria District Medical Society, Illinois.*—An admirable spirit of activity and progress appears, of late, to have been awakened among the Profession of the West. Local societies have been formed in different sections, which are well attended, and their proceedings are characterized by zeal and judgment. We have recently received, through the kindness of a friend, an account of the meeting of the District Society of Peoria, Illinois. Thirty members were present. An Annual Address was delivered, which was followed by a lecture by one of the members, and a report on the use of ether in obstetrical practice. Committees were appointed on medical inquiry, medical statistics, indigenous botany, on the formation of a library and museum, and on natural history, mineralogy and geology, to report at a subsequent meeting. Special committees on the "influence of a malarious atmosphere in the prevention and cure of pulmonary diseases," and on the utility of anaesthetic agents, were also appointed.

A good dinner, provided by the medical gentlemen of Peoria, is chronicled among the proceedings. This, albeit it may be considered last *and* least on such occasions, should not, in our humble opinion, be omitted, inasmuch as social enjoyments, properly regulated, contribute, in no small degree, to promote mutual acquaintance and good feeling. Some of the

District Societies in this State, we fancy, would be improved by the enthusiasm of our western brethren.

